# Relevance of Mediterranean diet and glucose metabolism for nephrolithiasis in obese subjects

**DOI:** 10.1186/1479-5876-12-34

**Published:** 2014-02-06

**Authors:** Laura Soldati, Simona Bertoli, Annalisa Terranegra, Caterina Brasacchio, Alessandra Mingione, Elena Dogliotti, Benedetta Raspini, Alessandro Leone, Francesca Frau, Laila Vignati, Angela Spadafranca, Giuseppe Vezzoli, Daniele Cusi, Alberto Battezzati

**Affiliations:** 1 Department of Health Sciences, University of Milan, Milano, Italy; 2 International Center for the Assessment of Nutritional Status (ICANS), Department of Food, Environmental and Nutritional Sciences (DeFENS), University of Milan, Milano, Italy; 3 Nephrology and Dialysis Unit, San Raffaele Hospital, Milan, Italy

## Abstract

**Background:**

Nephrolithiasis is more frequent and severe in obese patients from different western nations. This may be supported by higher calcium, urate, oxalate excretion in obese stone formers. Except these parameters, clinical characteristics of obese stone formers were not extensively explored.

**Aims:**

In the present paper we studied the relationship between obesity and its metabolic correlates and nephrolithiasis.

**Materials and methods:**

We studied 478 Caucasian subjects having BMI ≥ 25 kg/m^2^. The presence of nephrolithiasis, hypertension, diabetes mellitus and metabolic syndrome were noted. They underwent measurements of anthropometry (BMI and waist circumference, body composition), serum variables (fasting glucose, serum lipids and serum enzymes) and Mediterranean diet (MedDiet) nutritional questionnaire.

**Results:**

45 (9.4%) participants were stone formers. Subjects with high serum concentrations of triglycerides (≥150 mg/dl), fasting glucose (> 100 mg/dl) and AST (>30 U/I in F or >40 U/I in M) were more frequent among stone formers than non-stone formers.

Multinomial logistic regression confirmed that kidney stone production was associated with high fasting glucose (OR = 2.6, 95% CI 1.2-5.2, P = 0.011), AST (OR = 4.3, 95% CI 1.1-16.7, P = 0.033) and triglycerides (OR = 2.7, 95% CI 1.3-5.7, P = 0.01).

MedDiet score was not different in stone formers and non-stone formers. However, stone formers had a lower consumption frequency of olive oil and nuts, and higher consumption frequency of wine compared with non-stone formers.

**Conclusions:**

Overweight and obese stone formers may have a defect in glucose metabolism and a potential liver damage. Some foods typical of Mediterranean diet may protect against nephrolithiasis.

## Introduction

Dietary habit is generally acknowledged as a determinant in the pathogenesis of nephrolithiasis [1]. In this regard, many studies have shown a correlation between obesity and nephrolithiasis occurrence and severity, but were unable to clarify the mechanism leading to these relationships [2]. In addition, clinical pattern of obese stone formers has not been extensively studied. Nephrolithiasis risk could be exacerbated in obese subjects, because urine defects predisposing to stones are more striking in these patients. Higher excretion of calcium, uric acid and oxalate and lower urine pH and citrate excretion have been described in patients with metabolic syndrome and high body mass index (BMI) [3-6]. Insuline resistance and hyperinsulinemia were observed in overweight patients and may contribute to increase calcium and post-prandial oxalate excretion, thus, favoring calcium-oxalate stone formation [7].

Therefore, to clarify the relationship between obesity and nephrolithiasis, we analyzed in the present work the metabolic and clinical characteristics of obese and overweight subjects with a stone history and compared them with those without stones. We also analyzed the adherence of these subjects to Mediterranean diet with a dedicated questionnaire and tested the adherence to this diet by stone formers and non-stone formers with high BMI.

## Materials and methods

### Sample

Four hundred and ninety subjects (M/F 155/335, age 49 ± 10.3 yrs, Body weight 86.1 ± 16.42 kg, BMI 31.5 ± 4.99 kg/m^2^) were recruited among patients who attended the International Center for the Assessment of Nutritional Status (ICANS), University of Milan. Eligible for the study were Caucasian subjects of both genders, aged between 30 and 70 years and having BMI ≥ 25 kg/m^2^. They had to have no endocrine, neoplastic and inflammatory diseases, with the exception of type 2 diabetes mellitus and arterial hypertension, and their serum creatinine had to be ≤ 1.25 mg/dl. They had to take no chronic therapy with the exception of therapy for arterial hypertension, dyslipidemia and oral antidiabetics. Participants were studied for their nutritional status, blood variables and abdominal sonography. Blood variables were measured in participants after an overnight fast.

The presence of kidney stones was assessed by a self-questionnaire including 12 multiple-choice questions on kidney stones history based on sonographic exams and previous symptoms. The study protocol was approved by the Ethical Committee of the Milan University. All subjects gave informed consent to participate to the study.

### Nutritional status assessment

#### Anthropometric measurements

BMI (kg/m^2^) was calculated from body weight (kg) and height (m) using the formula: BMI = weight/height^2^. Waist and hip circumferences were measured as proposed by Lohman et al. [8] and waist-to-hip ratio was calculated to assess abdominal fatness. The same operator measured these variables according to conventional criteria and procedures [8]. Patients with BMI > 30 kg/m^2^ were defined as obese. Patients with BMI between 25 and 30 kg/m^2^ were defined as overweight.

### Ultrasound evaluation

Abdominal ultrasonography was performed with Logiq 3 Pro with a 3.5 MHz convex-array probe and 7.5 MHz linear probe. We measured the subcutaneous abdominal tissue as the distance between the epidermidis and external face of the rectus abdominis muscle and the visceral abdominal tissue width as the distance between the anterior wall of the aorta and the posterior surface of the rectus abdominis muscle measured above the umbilicus at the xiphoumbilical line [9]. The intra-examination coefficient of variation of the method was 0.8%.

### Clinical and biochemical parameters

The use of medications for the treatment of hypertension, diabetes and dyslipidemia was investigated.

Blood pressure was measured according to JNC-7 guidelines [10]. The hypertension diagnosis was based either on blood pressure measurements during clinical examination and subsequently confirmed in the following weeks, or on patients’ history and use of antihypertensive drugs.

Creatinine, uric acid, glucose, alanine transaminase (ALT), aspartate transaminase (AST) and γglutamyl transpeptidase (γGT), TSH, triglycerides, cholesterol, LDL, HDL were measured in serum.

### Mediterranean dietary pattern

Adherence to MedDiet pattern was assessed using a validated 14-item questionnaire with multiple-choice designed for this purpose [11]. Considered items were: olive oil (2 items), vegetables, fruits, wine, red meat, fish, sugar drinks, butter, legumes, backed products, white meat, nuts and sofrito (sauce made with tomato and onion, leek, or garlic and simmered with olive oil). For each answer was attributed a score from 0 to 14. Subjects with score ≥9 were considered to comply with MedDiet according to the median value as used in analyses of the PREDIMED group [12]. The questionnaire has been considered valid only when all 14 items were completed.

### Statistical analysis

Continuous variables were expressed in the text as mean ± standard deviation and compared by one-way analysis of variance (ANOVA) in stone formers and stone free patients and Tuckey test for comparisons between groups. Distribution of discrete variables was assessed using chi-square. The association of variables with nephrolithiasis was tested by multinomial logistic regression including sex, BMI (3 groups), total cholesterol, fasting glucose, HDL, triglycerides and blood pressure (2 groups). Odds ratio (OR) with 95% confidence interval (95% CI) was calculated. Statistical analysis was two tailed and was conducted at a 0.05 level. It was performed with SPSS 21 statistical package.

## Results

### Clinical variables

All 490 participants to the study had BMI ≥ 25 kg/m^2^. Forty-five participants were stone formers (9.2%), whereas 445 had no history of kidney stones. Among non-stone former 30 received therapy for dyslipidemia, 99 for hypertension and 9 for diabetes. Among stone former 7 received therapy for dyslipidemia, 16 for hypertension and 3 for diabetes. Stone formers were older than non-stone formers and had higher serum concentrations of triglycerides. A slight increase of fasting glucose and AST were also found (Table 1). Considering the distribution of these alterations (Table 2), subjects with high serum concentrations of triglycerides, fasting glucose and AST were more frequently found in stone formers than stone-free subjects.

**Table 1:** Serum variables in stone formers and non-stone formers

	**Stone formers**	**Non-stone formers**
N (M/F)	45 (18/27)	445 (137/308)
Age (years)	53 ± 10.9*	48 ± 10.2
Body weight (kg)	86.6 ± 15.01	86 ±16.57
BMI (kg/m^2^)	31.8 ± 5.12	31.5 ± 4.98
Total serum cholesterol (mg/dl)	211 ± 39.5	214 ± 38.4
Serum HDL (mg/dl)	53 ± 14.5	56 ± 14.4
Serum LDL (mg/dl)	133 ±39.3	138 ± 33.4
Serum triglycerides (mg/dl)	146 ± 110.5¶	110 ± 67.1
Fasting serum glucose (mg/dl)	102 ± 21.5†	96 ± 14.2
Serum uric acid (mg/dl)	5.2 ± 1.30	5.1 ± 1.39
Serum creatinine (mg/dl)	0.79 ± 0.186	0.77 ± 0.16
Serum TSH (ng/ml)	2.35 ± 1.268	2.23 ±1.49
Serum γGT (U/I)	39 ± 36.1	30 ± 32.7
Serum AST (U/I)	25 ± 12.6 †	21 ± 8.1
Serum ALT (U/I)	30 ± 19.6	26 ± 15.9
Waist circumference (cm)	105 ± 12.0	101 ± 12.7
Arm circumference (cm)	33 ± 4.4	34 ± 4.1
Wrist circumference (cm)	17 ± 1.4	17 ± 1.8
Subcutaneous abdominal tissue (cm)	3.2 ± 1.19	3.3 ± 1.12
Visceral abdominal tissue (cm)	6.8 ± 2.52	6.2 ± 2.58
Systolic blood pressure (mmHg)	132 ± 14.4	129 ± 13.7
Diastolic blood pressure (mmHg)	81 ± 9.3	80 ± 9.1
MedDiet score	7 ± 1.55	6.75 ± 1.63

*p = 0.002.

¶p = 0.044.

†p < 0.06.

**Table 2:** Frequency of subjects with alterated values of the explored variables in subjects with high BMI distinguished in stone formers and non-stone formers

	**Stone formers N (%)**	**Non-stone formers N (%)**
Subject count	45	445
Sex (M/F)	18/27	137/308
BMI > 30 kg/m^2^	24 (53.3)	233 (52.4)
Total serum cholesterol >200 mg/dl	27 (60)	276 (62.1)
Serum HDL <50 mg/dl in F or <40 mg/dl in M	31 (68.9)	339 (76.3)
Serum triglycerides >150 mg/dl	17 (37.7)‡	80 (17.9)
Fasting serum glucose >100 mg/dl	22 (48.8)*	121 (27.2)
Serum gGT > 24 U/I in F or >30 U/I in M	19 (45.2)	148 (36.5)
Serum AST >30 U/I in F or >40 U/I in M	7 (15.5)†	28 (6.3)
Serum ALT >35 U/I in F or >40 U/I in M	11 (24.4)	71 (16)
Waist circumference >80 cm in F or >92 cm in M	43 (95.6)	426 (95.7)
Subcutaneous abdominal tissue >4 cm	10 (22.2)	99 (22.9)
Visceral abdominal tissue >4 cm	40 (88.9)	346 (77.8)
Systolic blood pressure >130 mmHg	16 (35.5)	134 (30.1)
Diastolic blood pressure >85 mmHg	13 (28.9)	84 (18.9)
MedDiet score ≥ 9	8 (17.8)	67 (15.1)

‡p = 0.003.

*p = 0.004.

†p = 0.015.

Two hundred and fifty-seven subjects were obese (BMI ≥ 30 kg/m^2^): 9.3% of them were stone formers (n = 24). Two hundred and thirty-three subjects were overweight: similar to obese subjects, 9.0% of them were stone formers (n = 21). In both obese and overweight subjects, stone formers were older than non-stone formers.

Multinomial logistic regression confirmed that kidney stone production was associated with high fasting glucose (OR = 2.6, 95% CI 1.2-5.2, P = 0.011), AST (OR = 4.3, 95% CI 1.1-16.7, P = 0.033) and triglycerides (OR = 2.7, 95% CI 1.3-5.7, P = 0.01). A second multinomial logistic regression model also adjusted for VAT and SAT was used to investigate the association between kidney stone and abdominal obesity, confirming that kidney stone production was associated with high fasting glucose and triglycerides (data not shown).

The number of stone formers was higher in diabetic than non-diabetes patients (n = 6 [22.2% of diabetic patients] vs n = 39 [8.4% of non-diabetic patients], chi square = 5.8, DF = 1, p = 0.016). Conversely, stone disease was not associated with arterial hypertension (n = 15 [12.6% of hypertensive patients vs n = 30 [8.1% of normotensive patients]).

The analysis of non-diabetic subjects (n = 463) confirmed that stone formers were older than stone-free subjects (51 ± 10.3 vs 48 ± 10 yrs, p = 0.037). The number of patients with high fasting serum glucose (n = 16 [41% of stone formers] vs n = 97 [23.7% of stone free patients]; p = 0.019), AST (n = 6 [15.3%] vs n = 25 [5.9%]; p = 0.015) and tryglicerides (n = 16 [41%] vs n = 75 [17.7%]; p = 0.001) was higher in non-diabetic stone formers than stone-free subjects.

Among normotensive subjects (PA ≤ 130/80 mmHg, n = 371), stone formers were older than stone-free subjects (52 ± 11.2 [n = 30] vs 46 ± 9.7 [n = 341] yrs, p = 0.001). Subjects with high fasting serum glucose (n = 16 [53.3%], vs n = 76 [22.2%]; p = 0.001), AST (n = 6 [20%] vs n = 22 [6,5%]; p = 0.007) and tryglicerides (n = 13 [43.3%] vs n = 60 [17.6%]; p = 0.001) were more frequent in stone formers than stone free subjects.

The analysis of normotensive and non-diabetic subjects (n = 357) again showed that stone formers more frequently had high fasting serum glucose (n = 13 [48.1%], vs n = 66 [20%]; p = 0.001), AST (n = 5 [18.5%] vs n = 19 [5.8%]; p = 0.01) and tryglicerides (n = 12 [44.4%] vs n = 56 [17%]; p = 0.001).

### Mediterranean diet

Mediterranean diet adherence was evaluated with a specific questionnaire. In our population n = 75 subjects were adherent to Mediterranean diet (Medscore ≥9) with a mean score of 6.8 ± 1.62. MedDiet score was not different in stone formers and subjects with no stone. However, the analysis of food frequency in Medscore questionnaire showed that stone formers had lower consumption frequency of olive oil and nuts, but higher consumption of wine compared with stone-free subjects (Table 3). Multinomial regression analysis observed that decreased risk of stone was associated with olive oil consumption whereas it increased in association with wine consumption (Table 4).

**Table 3:** Analysis of food frequency in Medscore questionnaire in stone formers and non-stone formers

	**Stone formers N (%)**	**Non-stone formers N (%)**
N (M/F)	45	445
Use of olive oil as main culinary lipid	45 (100)	443 (97.3)
Olive oil ≥ 4 tablespoons	11 (24.4)	188 (42.5)*
Vegetables ≥ 2 servings/day	28 (62.2)	252 (56.6)
Fruits ≥3 servings/day	8 (17.8)	75 (16.9)
Red/processed meats <1/day	34 (75.5)	293 (65.8)
Butter, cream, margarine <1/day	45 (100)	431 (96.8)
Soda drinks <1/day	38 (84.4)	377 (84.7)
Wine glasses ≥7/week	27 (60)	138 (31)†
Legumes ≥3/week	2 (4.4)	17 (3.8)
Fish/seafoods ≥3/week	2 (4.4)	30 (6.7)
Commercial sweets and confectionery <3/week	30 (66.7)	236 (53)
Free nuts ≥1/week	0 (0)	51 (11.5)*
Poultry more than red meats	29 (64.4)	259 (58.2)
Use of sofrito sauce ≥2/week	23 (51.1)	245 (55.1)

Items included in the MedScore questionnaire. Between parentheses there is the response showing adherence to Mediterranean diet.

Number (percentage) of subjects satisfying criteria of Mediterranean diet for each item [11,12].

*p = 0.019.

†p = 0.001.

**Table 4:** Multinomial regression analysis of olive oil and wine consumption in stone formers and non-stone formers

	**Score 1 <1 spoon/day**	**Score 2 1-3 spoons/day**	**Score 3 or 4 ≥4 spoons/day**	
Olive oil				
Stone formers	6 (13.3)	28 (62.2)	11 (24.4)	
Non-stone formers	33 (7.5)	221 (50)	188 (42.6)	
OR	1	0.7	0.32	
95% CI		0.3-1.8	0.1–0.9	
p		0.46	0.036	
	Score 1	Score 2	Score 3	Score 4
	No consumption	1–2 glasses/week	3–7 glasses/week	>7 glasses/week
Wine				
Stone formers	13 (30.2)	4 (9.3)	16 (37.2)	10 (23.3)
Non-stone formers	189 (42.9)	115 (26.1)	83 (18.8)	54 (12.2)
OR	1	0.51	2.8	2.7
95% CI		0.2–1.6	1.3–6.1	1.1–6.5
p		0.24	0.009	0.027

Olive oil and wine consumption was quantified according to the criteria adopted by the MedScore questionnaire that attributed a score between 0 and 4 to these item. Due to the low patient number, the score 3 and 4 of olive oil consumption were taken together in this multinomial regression analysis.

Considering only subjects without diabetes (n = 460), it was confirmed that stone formers consumed lower olive oil and nuts and greater quantity of wine compared with stone-free subjects: 10 (25.6%) stone formers vs 176 (41.5%) stone-free subjects consumed ≥4 spoons of olive oil in a day (p = 0.05); 23 (59%) stone formers and 130 (30.7%) stone-free subjects drank ≥7 glasses in a week (p = 0.001); no stone formers, but 49 (11.6%) stone free subjects ate nuts (p = 0.028).

## Discussion

The present work analyzes the metabolic pattern of overweight and obese stone formers (BMI ≥ 25 kg/m^2^) to find differences related to the kidney stone disease. Contrarily to previous studies, high body weight was not positively associated with kidney stones in our patients [13-15]. We found that the serum concentration of AST, fasting glucose and triglycerides were higher in stone formers, suggesting that stone production may be associated with an altered handling of glucose and triglycerides and with a liver disorder. Abdominal obesity (both VAT and SAT components) seems not to be associated with stone production. Insuline resistance and hyperinsulinemia have been reported in subjects with high BMI [16] and, although not explored in our patients, these defects could sustain the observed serum alteration of glucose and triglycerides. Iperinsulinemia may contribute to hyperexcretion of calcium and oxalate, but also decrease urine pH by inhibition of ammoniagenesis in proximal tubular cells, thus predisposing patients to uric acid stones [3]. From a metabolic point of view, triglycerides and glucose are important energy reserve and the increase of their serum levels may favor the raise of visceral fat, while products of their catabolism may increase the acid load for the body that may decrease citrate excretion [2,3]. Also, a diet rich in animal fats and proteins has been involved in kidney stone production because it causes acid overload and urine acidification [17-19].

The increase of serum AST might be expression of a liver damage that could be supported by a fat accumulation leading to the condition of hepatic steatosis caused by unhealthy eating habits in overweight-obese patients. An abnormal visceral and subcutaneous fat accumulation was found to be associated with hepatic steatosis in subjects without nephrolithiasis [20]. However, the higher consumption of red wine among the stone formers may have influenced the outcome. Therefore, further studies confirming the association between kidney stones and abnormal liver enzymes, are needed.

Subjects included in the present study showed low adherence to Mediterranean Diet without difference coupled to nephrolithiasis. The analysis of single items characterizing MedDiet in the questionnaire showed that the consumption of olive oil and nuts were lower in stone formers, suggesting a possible higher intake of animal fats in these patients inducing an urine environment that predisposes to kidney stone production as previously reported [17-19]. Positive effects of olive oil were evidenced in previous studies observing the association at its dietary use with high HDL serum levels and low blood pressure, LDL serum levels and obesity [12,21-23]. Although these advantages, its potential protective effect against nephrolithiasis in subjects with high BMI remains unexplained. Future prospective studies are needed to better understand the protective role of olive oil in the pathogenesis of nephrolithiasis. These findings are consistent with previous prospective surveys analyzing DASH diet effects in stone forming patients [24,25]. On the contrary, the negative association of wine with stone risk has not been previously recorded because prospective surveys showed that wine, similar other beverages, reduces the risk of stones [26,27]. This discrepancy may be explained by our choice to study only obese and overweight subjects and suggests that wine may have different effects on the lithogenesis according to the patient substrate. Specific studies are needed to confirm this result in obese subjects. Similar consideration could be proposed for legumes that for unknown reason resulted predisposing to stones in our population.

Limitations of this study are the fact that we could not distinguish patients with different stone composition and that the presence of stones was self-reported by patients. In addition, our study is cross-sectional and not prospective and therefore it cannot provide any conclusive causal inference from its findings. Another limitation of our study is the fact that we did not measure urine calcium, urate and oxalate, and we did not investigate the presence of liver steatosis with liver ultrasonography or biopsy in our patients. However, the aim of our study was to evaluate parameters of metabolism used in the clinical routine and the attitude to Mediterranean diet.

In conclusion, our findings suggest that overweight and obesity could be associated with kidney stones through a defect in glucose metabolism. Furthermore, obese stone formers may be characterized by a potential liver damage. In our knowledge, these alterations have not been previously described in stone formers. Finally, some foods typical of Mediterranean diet may protect subjects with high BMI against the risk of nephrolithiasis.
